# Statistical Models for Detecting Differential Chromatin Interactions Mediated by a Protein

**DOI:** 10.1371/journal.pone.0097560

**Published:** 2014-05-16

**Authors:** Liang Niu, Guoliang Li, Shili Lin

**Affiliations:** 1 Department of Statistics, The Ohio State University, Columbus, Ohio, United States of America; 2 National Key Laboratory of Crop Genetic Improvement, Center for Systems Biology, Center for Bioinformatics, College of Life Science and Technology, Huazhong Agricultural University, Wuhan, Hubei, China; University of Southern California, United States of America

## Abstract

Chromatin interactions mediated by a protein of interest are of great scientific interest. Recent studies show that protein-mediated chromatin interactions can have different intensities in different types of cells or in different developmental stages of a cell. Such differences can be associated with a disease or with the development of a cell. Thus, it is of great importance to detect protein-mediated chromatin interactions with different intensities in different cells. A recent molecular technique, Chromatin Interaction Analysis by Paired-End Tag Sequencing (ChIA-PET), which uses formaldehyde cross-linking and paired-end sequencing, is able to detect genome-wide chromatin interactions mediated by a protein of interest. Here we proposed two models (One-Step Model and Two-Step Model) for two sample ChIA-PET count data (one biological replicate in each sample) to identify differential chromatin interactions mediated by a protein of interest. Both models incorporate the data dependency and the extent to which a fragment pair is related to a pair of DNA loci of interest to make accurate identifications. The One-Step Model makes use of the data more efficiently but is more computationally intensive. An extensive simulation study showed that the models can detect those differentially interacted chromatins and there is a good agreement between each classification result and the truth. Application of the method to a two-sample ChIA-PET data set illustrates its utility. The two models are implemented as an R package *MDM* (available at http://www.stat.osu.edu/~statgen/SOFTWARE/MDM).

## Introduction

Chromatin interactions mediated by a protein of interest are of great scientific interest [1]–[4]. Such interactions can have different intensities in different types of cells or in different developmental stages of a cell. The differences of the interaction intensities can be associated with a disease or with the development of a cell. For example, a study [5] on prostate cancer found that the *UBE2C* oncogene interacts with two distal binding sites of Androgen Receptor (AR, an important protein in prostate cancer) only in Castration-Resistant Prostate Cancer (CRPC) cells but not in Androgen-Dependent Prostate Cancer (ADPC) cells. These two interactions are required for the progression from ADPC to CRPC. Another study [6] on the maturation of mouse thymocytes showed that protein Runx1 binds to a silencer of gene *CD4*, and thus represses *CD4* expression, only in the early stages of mouse thymocytes. Such repression is crucial in the mouse thymocyte maturation. Also, a study [7] on the human *β*-globin genes found that the interactions between the *β*-globin locus control region (LCR) and *β*-globin genes, which are mediated by CCCTC-binding insulator protein (CTCF) on human chromosome 11, are stronger in erythroid cells than in non-erythroid cells. These differential interactions make the transcription of *β*-globin genes active only in erythroid cells. Another study [8] on the proto-oncogene *Kit*, which encodes a receptor tyrosine kinase that is essential for normal hematopoiesis, found that the GATA-2 mediated interaction between a distal enhancer and the gene promoter in the immature cells disappears upon cell maturation. Such a change makes the gene expression downregulated upon cell maturation. Therefore, detecting differential chromatin interactions mediated by a protein is of significant value in deciphering the development of complex diseases such as cancer or the development of a cell.

The study of chromatin interactions mediated by a protein has been aided by Chromatin Interaction Analysis by Paired-End Tag Sequencing (ChIA-PET) [9], a recent molecular technique derived from Chromosome Conformation Capture (3C) [10]. Unlike Hi-C [11], another derivative of 3C, which detects genome-wide chromatin interactions, ChIA-PET can be used to investigate chromatin interactions mediated by a specific protein in a genome-wide manner. Here is a schematic description of ChIA-PET: first, the chromatin is cross-linked with formaldehyde and then sonication is employed to break up the chromatin; second, specific antibody of choice is used to precipitate the chromatin fragments bound by the protein of interest (Chromatin Immunoprecipitation, or ChIP) and the biotinylated oligonucleotide half-linkers containing flanking MmeI (a restriction enzyme) sites are used to ligate the tethered DNA fragments; third, the linkers are ligated and MmeI is introduced to digest the ligated fragments; finally, the ligation products are purified using streptavidin-coated beads and the pair end tags are sequenced through high throughput paired-end sequencing.

A ChIA-PET experiment generates millions of paired-end sequencing reads that can be mapped to the reference genome [9]. Pairs of DNA regions, which we refer to as fragment pairs, can then be determined from the read pairs. Those chromatin interactions with different intensities can then be inferred from the different frequencies of the fragment pairs in ChIA-PET experiments on different cells. However, such inference is not straightforward. The reason is that fragment pairs determined from ChIA-PET experiments include both signal and noise, i.e., fragment pairs that are present due to interactions, termed true pairs, and those that are present due to their close proximity during the ligation step, termed false pairs or random collisions. Thus, an ideal method should be able to distinguish the true pairs from the false pairs, and to detect the differences of interaction intensities.

In this paper we propose a *Two-Step Model* and a *One-Step Model* to detect chromatin interactions with different intensities from two sample ChIA-PET count data mediated by the same protein. The Two-Step Model proceeds in two steps: first, true pairs are distinguished from false pairs in each sample by the model MC_DIST [12]; second, the pairs that are classified as true pairs in both samples are further investigated using a mixture modeling framework and those with different interaction intensities are determined. The One-Step Model combines the two steps in the Two-Step Model to make use of the data more efficiently, although it typically entails greater computational burden. An extensive simulation study was carried out to evaluate, compare, and contrast their performance. We applied the more efficient One-Step model to analyze a two-sample ChIA-PET data set. Our findings appear to be consistent with those in the literature. An R package implementing the above two models is available at http://www.stat.osu.edu/~statgen/SOFTWARE/MDM.

## Methods and Analysis

Let 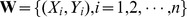 be a set of two-dimensional random vectors, where 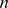 is the total number of fragment pairs, and 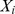 (or 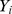) represents the count of fragment pair 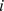, i.e., the number of read pairs mapped to the 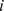-th pair of fragments in a ChIA-PET experiment performed for sample one (or two). We require that 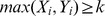, where 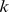 (if 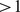) is the cut-off value used in a non-analytical preprocessing step to weed out random collisions. For instance, in [9], 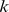 was set to be 2, as it was believed that a fragment pair observed only once in both samples was a false pair and should be excluded from further analysis. This threshold can be set by investigators based on their knowledge of the experiment.

Our objective is to identify the fragment pairs with different interaction intensities in the two samples. Such fragment pairs include those with interactions only in one sample but not in the other sample. Specifically, we want to classify the fragment pairs into the six categories enumerated in Table 1. The pairs in categories 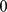, 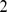, 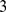 and 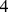 are of interest, as categories 0 and 2 contain true pairs with different interaction intensities in the two samples and categories 3 and 4 contain pairs with interactions, i.e., true pairs, only in one of the two samples. The other two categories are not of interest, as category 1 contains pairs with same interaction intensities in both samples, and category 5 contains pairs with no interactions, i.e., false pairs, in both samples.

**Table 1:** Category of pairs.

category	sample one^a^	sample two^b^	interaction intensity^c^
0	true	true	decrease
1	true	true	same
2	true	true	increase
3	true	false	decrease
4	false	true	increase
5	false	false	NA

a Whether the pair is a true pair or a false pair in sample one.

b Whether the pair is a true pair or a false pair in sample two.

c The change of the interaction intensity of the pair from sample one to sample two: either increase, decrease or (stay the) same.

To achieve the above objective, we first normalize the observed count data to make the counts comparable across the two samples. Assuming that, without loss of generality, sample one has a smaller total count than sample two does, the normalization is done simply by multiplying all the counts in sample one by a factor *c* and then rounding the results to their nearest integers, where *c* is the ratio of the total count of sample two to the total count of sample one. We then apply either a *Two-Step Model* or a *One-Step Model* on the normalized data (still denoted by 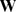 for the sake of brevity) to classify the fragment pairs into the six categories. The two models are discussed in details in the following two subsections.

### Two-step Model

In this model, the first step is to apply MC_DIST on the count data for each sample separately to determine whether a pair is a true pair or a false pair in the sample. MC_DIST is a Bayesian mixture model with two (pair-specific) truncated Poisson components, and each component models the count conditional on the pair being a true or a false pair. The pair-specific mixing proportion (weight) is assumed to follow a beta distribution whose parameters are determined by the characteristics of the pair such as the *marginal count* and the *distance*. Briefly, the marginal count of a particular pair is defined as the total number of pairings that contain either fragment of the pair; the distance of a particular pair refers to how close the pair of fragments are to important landmarks of the genome such as Transcription Factor Binding Site (TFBS) and Transcription Start Site (TSS). These definitions will be elaborated below. The reason for modeling the weight in such a way is to incorporate the data dependency and the extent to which the locations of the pair relative to known DNA loci to leverage additional information. At the conclusion of step one, all pairs are divided into three groups: pairs that are classified as false in both samples, pairs that are classified as false in one sample but are classified as true in the other sample, and pairs that are classified as true in both samples. The pairs in the first two groups are then classified into category 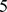, 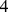 and 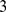 accordingly. The pairs in the last group are passed to the second step to be further classified into categories 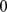, 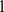 or 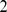. Without loss of generality, we assume that these pairs are labeled 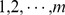. Note that both 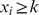 and 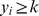 as a pair with count less than 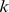 is screened out before applying the first step.

The second step of the Two-Step Model is to apply a three-component mixture model to the pairs with indices 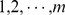 to classify those pairs into categories 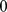, 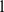 or 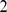. Specifically, we assume that
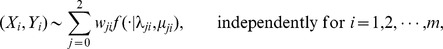
where 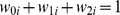; 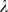
’s and 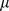
’s represent interaction intensities and are assumed to be under several constraints for identifiability, which are discussed later in details. The component probability mass function 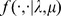 is a product of two 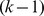-truncated Poisson distributions:



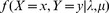





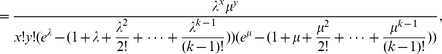
for 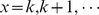 and 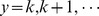. The use of the (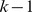)-truncated Poisson distributions is motivated by the fact that 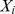 and 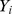 are counts and that they are both at least 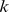, as discussed above.

The constraints on 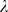
’s and 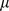
’s are:
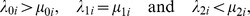
for 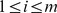. The constraints are imposed as such to make the mixture component with indices 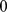, 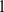 and 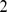 correspond respectively to category 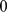, 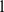 and 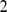. Since 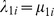, we only include 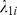 in the following discussion.

It is easy to see that the counts of two pairs with a common fragment are correlated with each other within the sample and therefore the data are dependent within the sample. To take such data dependency into account, we cast the above model into a Bayesian framework and incorporate the data dependency into the prior distributions of 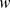
’s. The details of the Bayesian framework are described as follows. We assume
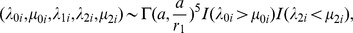
independently for 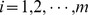, where 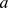 is the shape parameter of the gamma model, and 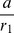 is the rate parameter (thus the mean of 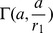 is 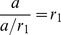). This is a product of five independent gamma distributions conditional on 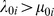 and 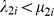. For the hyperparameters 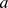 and 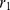, we assume that they are independent and



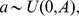





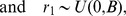
where 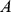, 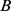 are large constants so that the priors are uninformative. In the simulation study and the application, we choose 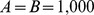.

To incorporate the data dependency into the modeling, we define 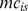, or *marginal count*, for pair 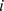 (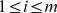) in sample 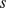 (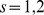) as the sum of two *marginal counts* of the two fragments of the pair in the sample, where the marginal count of a fragment is defined as the sum of the counts of all pairs containing that fragment. The marginal counts of the pairs reflect the dependency among the data within the sample. Note that the marginal count of a pair is defined and used in the same way as in MC_DIST. Using the two marginal counts of pair 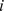 in the two samples, we model the 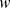
’s for pair 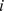 by a Dirichlet distribution as follows:
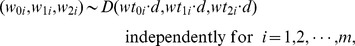
where 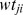 are constants defined as follows:



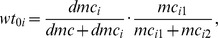





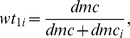





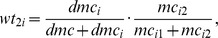
and 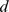 is a hyperparameter. Here 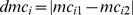 and 
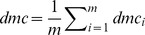
. The 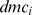 stands for “difference of marginal counts of pair 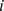 (between the two samples)”. The motivation for specifying such a prior distribution is that the larger the 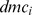 is, the more likely pair 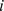 has different interaction intensities between the two samples. We also assume that 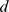 follows a uniform distribution, i.e., 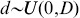, where 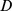 is a large constant. In the simulation study and the application, we choose 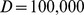. Note that 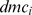 can be zero for some pairs, in those cases we replace the quantity by a small value such as 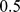 to avoid the corresponding parameter of the Dirichlet distribution being zero.

To determine the category of pair 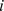, we define a latent discrete random variable 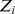 such that:
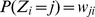
and



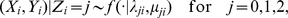
independently for 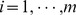. The 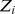 is an indicator variable in which 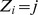 implies that the pair 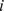 is in category 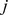, where 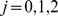. We conclude that pair 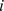 is in category 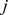 whenever 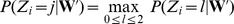, where 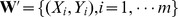. The posterior probabilities are calculated by a Markov chain Monte Carlo (MCMC) method. Specifically, in each iteration, 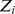 and 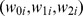 are sampled from their own full conditional distributions (discrete distribution for 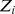 and Dirichlet distribution for 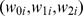). On the other hand, since the full conditional distributions of the other parameters are not of a known form, we utilize the Metropolis-Hastings algorithm using either a log-normal distribution (for 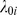, 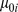, 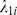, 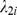, 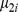, 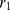 or 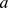) or a uniform distribution (for 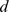) as the proposal distribution.

The above Two-Step Model is computationally efficient since it only considers a subset of the observed data in step two, i.e., only those pairs that are concluded to be true in both samples in step one. However, the trade-off for such computational efficiency is its ignorance of potential classification errors in step one. As such, we propose the following One-Step Model as a remedy, which, however, is more computationally intensive.

### One-step Model

In this model, we assume that 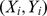 follows a six-component mixture distribution:
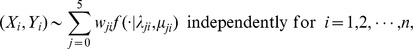
where 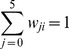, and 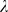
’s and 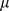
’s represent the interaction intensities and are assumed to be under several constraints for identifiability, as discussed below. The component probability mass function 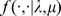 is the conditional joint distribution for two independent Poisson random variables given that at least one of them is at least 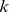, i.e.,



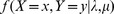





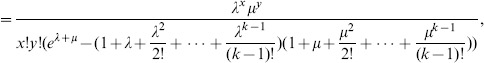
for 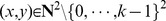, where 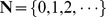.

The constraints on the 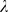
’s and 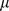
’s are:
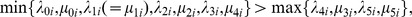





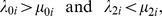
for all 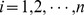. The constraints are imposed as such to make the mixture components with indices 0 to 5 correspond respectively to category 0 to 5: the first constraint comes from a biologically supported assumption [13] that we expect the interaction intensity to be higher for a true pair, and the other two constraints comes from the nature of category 0 and category 2. Note that the last two constraints are the same as the constraints on the 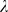
’s and 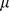
’s in the mixture model of the second step of the Two-Step Model.

In addition to the data dependency that we discussed above, the genomic locations of each pair relative to known DNA loci of interest are also informative for our classification purpose. To include the above two data features into the model, we cast the above model into a Bayesian framework and make use of both data features to specify the prior distributions of 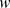
’s. The details of the Bayesian framework are described as follows.

We assume that
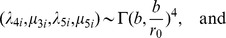





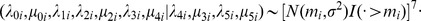





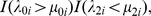
independently for 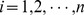, where 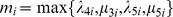, and 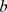 and 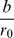 are respectively the shape parameter and the rate parameter of the gamma distribution (thus the mean of 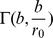 is 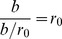). For the hyperparameters 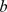, 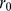 and 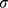, we assume that they are independent and,



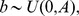





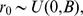





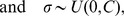
where 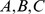 are large constants so that such prior distributions are uninformative. In the simulation study and the application, we choose 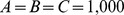.

To take the data dependency and the genomic locations of the pairs relative to known DNA loci into account, we make use of two pieces of additional data, the *marginal count* and *distance*, for each pair and use them to specify the priors of the 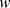
’s. The marginal count of a pair is as defined in the previous subsection to reflect data dependency. The distance of a pair is defined as follows and it measures the extent to which the locations of the pair relative to a pair of known DNA loci of interest. For pair 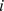, the distance 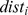 is the minimum of the following two values: the sum of the distance between the midpoint of 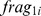 and the nearest midpoint of a Transcription Factor Binding Site (TFBS) of protein 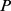 and the distance between the midpoint of 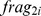 and the nearest gene Transcription Start Site (TSS); the sum of the distance between the midpoint of 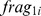 and the nearest gene TSS and the distance between the midpoint of 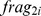 and the nearest midpoint of TFBS of protein P. Here 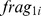 and 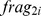 are the two fragments of pair 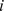 and 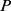 is the protein of interest in the ChIA-PET experiments. In essence, 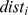 measures the extent to which pair 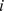 is related to a pair of DNA loci of interest for looping, one being TFBS and the other being TSS. Note that the distance is defined in such way to encourage anchoring of the pair by TFBS and TSS and is used in the same way as in MC_DIST.

With 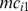, 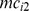 and 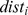 for pair 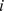, we model 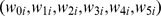 by a Dirichlet distribution:
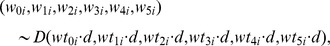
independently for 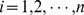, where 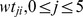 are constants defined as:



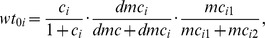





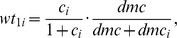





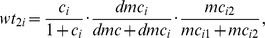





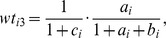





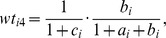





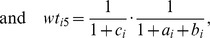
and 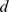 is a hyperparameter. In the above equations, 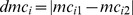 and 
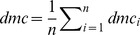
 (as in the previous section); 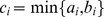 where 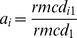 and 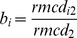. Here 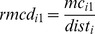, 
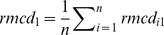
; 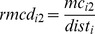, 
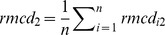
. The 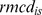 stands for the “ratio of the marginal count to the distance of pair 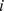 in sample 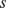
”. The motivations for specifying such a prior distribution are as follows. First, the larger the 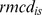 is, the more likely pair 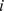 is a true pair in sample 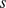. This is because that the larger 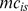 is, the more active the two fragments of pair 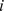 are, thus pair 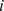 is more likely to be a true pair in sample 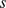; and that the smaller 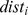 is, the more likely the pair is observed because of the interaction between the nearby TFBS and the nearby gene TSS. Second, the larger the 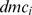 is, the more likely the interaction intensities of pair 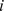 are different in the two samples. We also assume that 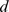 follows a uniform distribution, i.e., 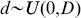, where 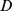 is a large constant and should be large enough to balance the difference between *mc* and *dist*, since they are in different units. In the simulation study and the application, we choose 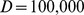. Note that 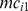, 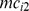 and 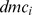 can be zero for some pairs, in those cases we replace the quantity by a small value such as 0.5 to avoid the corresponding parameter of the Dirichlet distribution being zero.

To determine to which category pair 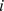 belongs, we define a latent discrete variable 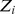 such that
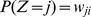
and



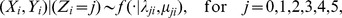
independently for 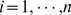. The 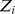 is an indicator variable in which 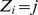 implies that pair 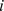 is in category 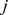. We conclude that pair 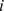 is in category 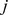 whenever 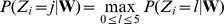, The posterior probabilities are calculated by a Markov chain Monte Carlo (MCMC) method. Specifically, in each iteration 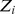, 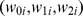 and 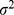 are sampled from their own full conditional distributions (discrete distribution for 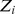, Dirichlet distribution for 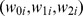 and inverse gamma distribution for 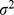). On the other hand, since the full conditional distributions of the other parameters are not of a known form, we utilize the Metropolis-Hastings algorithm using either a log-normal distribution (for 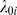, 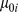, 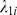, 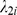, 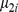, 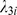, 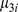, 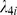, 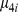, 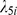, 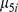, 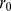 or 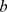) or a uniform distribution (for 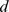) as the proposal distribution.

Compared with the Two-Step Model, the above One-Step Model models the count data from two samples jointly and thus makes use of data more efficiently. It also takes the data dependency and the relevance of each pair to known DNA loci into account. Thus it is expected to make more accurate classifications than the two-Step Model does. The trade-off is its greater computational intensity.

### Simulation Study

#### Fragment library

We generated a library of 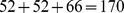 DNA fragments cut by HindIII (a restriction enzyme) in the human reference genome (hg19). Specifically, 52 of the fragments contain only one AR binding site and no Octamer transcription factor 1 (OCT1) binding site; 52 of the fragments contain only one OCT1 binding site and no AR binding site; the rest of the 66 fragments were randomly selected from the fragments with no AR or OCT1 binding site, three from each of the 22 autosomes. To facilitate later discussion, we call the above three groups of fragments as *group A*, group *group B*, and *group C*, respectively, and label the fragments as 1–52 (group A), 53–104 (group B) and 105–170 (group C).

The motivations for constructing such a fragment library are that we wanted to imitate two ChIA-PET experiments aiming to study genome-wide differential chromatin interactions bound by AR in two different prostate cancer cells, and that OCT1 is an important coregulator of AR in such cells [14]. The 52 fragments with AR binding sites (fragments in group A) were thus designated as fragments that mark TFBS, and the 52 fragments with OCT1 binding sites (fragments in group B) were designated as fragments that mark gene TSS’s. The midpoint of each OCT1 binding site on each group B fragment was designated as a gene “TSS”. We note that although our in-silico design does not match exactly to the ChIA-PET experiment, the data generated are similar in characteristics to the processed ChIA-PET data for analysis. For example, fragment pairs with true interactions have greater chance of ligations and thus larger counts of paired-end reads.

#### Pair library

**Figure 1:**
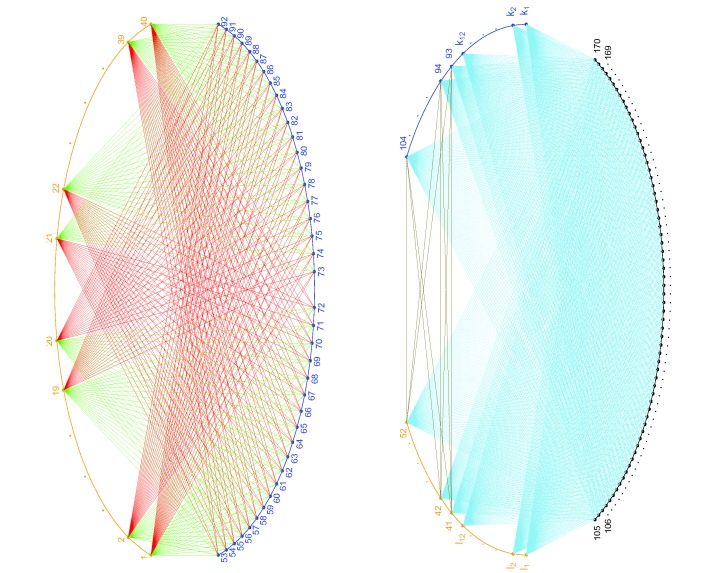
Illustration of sampling probabilities for simulated data set one. Left figure: Pair set one. All orange points (fragments 1–40) are connected to all blue points (fragments 53–92), either by green lines or by red lines. Fragments 1–20 and fragment 53–72, also fragments 21–40 and fragments 73–92, are connected by green lines. Other lines are red. All pairs in this graph were designated as true pairs in the simulation for data set one and the ratio of the sampling probabilities for a green line to that for a red line is 2:1. Right figure: Pair set two. All orange points (fragments 41–52 and fragments *l*_1_− *l*_12_) and all blue points (fragments 93–104 and fragments *k*_1_− *k*_12_), are connected to all black points (fragment 105–170) by sky-blue lines. Fragments 41–52 are connected to fragment 93–104 by gold lines. All pairs in this graph were designated as false pairs in the simulation for data set one and were assigned the same sampling probability. The ratio of the sampling probabilities for a green line, to that for a red line, and to that for a sky-blue/gold line is 6:3:1.

**Figure 2:**
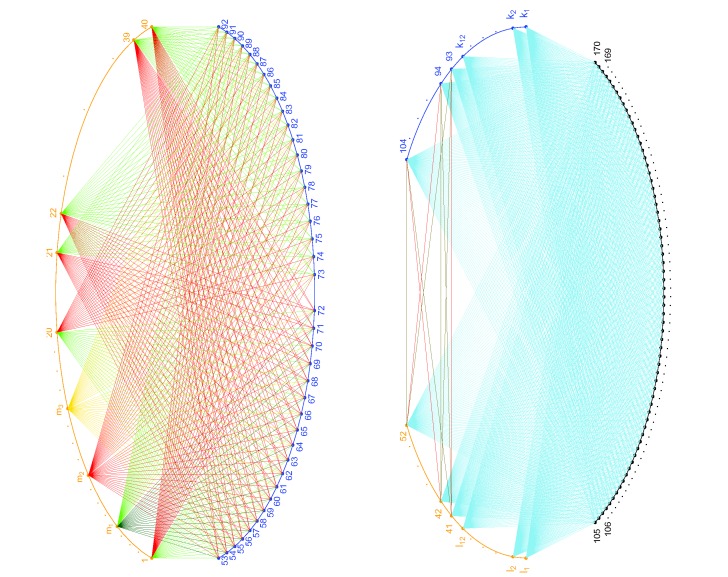
Illustration of sampling probabilities for simulated data set two. Left figure: Pair set one. All orange points (fragments 1–40) are connected to all blue points (fragments 53–92), either by dark green, green, red or gold lines. Fragments 1–20 (except 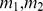 and 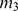) and fragment 53–72, also fragments 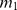, 21–40 and fragments 73–92, are connected by green lines. Fragment 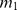 and fragments 53–72 are connected by dark green lines. Fragment 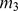 and fragments 53–92 are connected by gold lines. All other lines are red. The ratio of the four sampling probabilities for a dark green line, to that for a green line, to that for a red line, to that for a gold line is 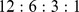. Right figure: Pair set two. All orange points (fragments 41–52 and fragments 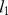
–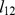) and all blue points (fragments 93–104 and fragments 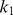
–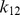), are connected to all black points (fragments 105–170) by sky blue lines. Fragments 41–52 are connected to fragments 93–104 either by gold or red lines. There are 10 red lines in the graph, with only 3 of them are shown for the sake of simplicity. The ratio of the sampling probability for a red line to that for a sky-blue/gold line is 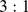. The sky-blue lines and gold lines (in both figures) represent false pairs and were assigned the same sampling probabilities.

We generated a library of 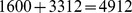 fragment pairs using the above fragment library. The first 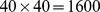 fragment pairs were obtained by pairing the 40 fragments with the smallest distances (defined as follows) in group A (with labels 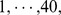 without loss of generality) with each of the 40 fragments with the smallest distances (defined as follows) in group B (with labels 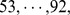 without loss of generality). Here the *distance* of a group A fragment is defined as the genomic distance between the midpoint of the fragment and the midpoint of the (unique) AR binding site on it, while the *distance* of a group B fragment is defined as the genomic distance between the midpoint of the fragment and the midpoint of the (unique) OCT1 binding site, i.e., the gene “TSS”, on it. The rest of the 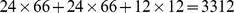 pairs were obtained as follows: We randomly chose 12 fragments (with labels 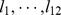) from fragments 1–40, and, together with fragments 41–52, paired them with the 66 group C fragments to obtain 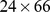 pairs. We also randomly chose 12 fragments (with labels 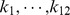) from fragments 53–92, and, together with fragments 93–104, paired them with the 66 group C fragments to get another 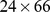 pairs. Finally, we paired the fragments 41–52 with the fragments 93–104 to get the remaining 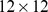 pairs. The pair library construction is illustrated in Figure 1 and also in Figure 2. To facilitate discussion, the set containing the first 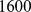 fragment pairs is hereinafter referred to as pair set one and the set of the rest 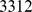 fragment pairs is hereinafter referred to as pair set two.

#### Sampling probabilities

We simulated two ChIA-PET count data sets using the above pair library, The sampling probabilities for each simulation are described as follows.

In the simulation for data set one, we set the sampling probabilities as follows. First, we divided the 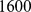 pairs in pair set one into two sets of equal size: a *true high* set and a *true low* set, shown as the green lines and red lines, respectively, in Figure 1. To do this, we randomly selected 20 fragments (with labels 1–20 without loss of generality) from fragments 1–40, and randomly selected 20 fragments (with labels 53–72 without loss of generality) from fragments 53–92, and used the pairs formed by fragments 1–20 and fragments 53–72, together with those formed by fragments 21–40 and fragments 73–92, to form the true high set. Other pairs in pair set one were used to form the true low set. Then, we set the sampling probabilities in such a way that the ratio of the sampling probability of a true high pair, to that of a true low pair, and to that of each of the rest 3312 pairs (pair set two), is 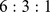. Thus for each pair in the three sets, the sampling probabilities are 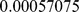, 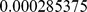 and 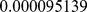, respectively. In this way, the pairs in set one were designated as true pairs and the pairs in set two were designated as false pairs in the simulation for data set one.

In the simulation for data set two, we set the sampling probabilities as follows. First, we randomly chose three fragments from fragments 1–20, with labels 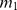, 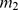 and 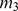. Second, we set the sampling probabilities of the 40 pairs formed by fragment 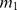 and fragments 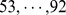 to be twice of their old sampling probabilities, i.e., either 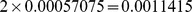 or 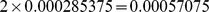. We set the sampling probabilities of the 20 pairs formed by fragment 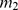 and fragments 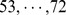 to be half of their old sampling probabilities, i.e., 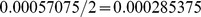. Further, we set the sampling probabilities of the 40 pairs formed by fragment 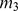 and fragments 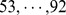 to be 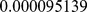, i.e., the sampling probability for a false pair in the simulation for data set one. Third, we randomly chose 10 pairs from the 144 pairs formed by fragments 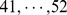 and fragments 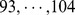, and set their sampling probabilities to be three times of their old sampling probabilities, i.e., 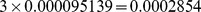. Finally, we set the sampling probabilities of the remaining 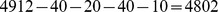 pairs in the pair library to be the same as their old sampling probabilities. In this way, the 40 pairs formed by fragment 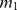 and fragments 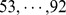 were designated as pairs in category 2; the 20 pairs formed by fragment 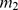 and fragments 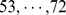 were designated as pairs in category 0; the 40 pairs formed by 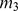 and fragments 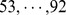 were designated as pairs in category 3; the 10 pairs that we randomly chose from pair set two were designated as pairs in category 4; and the remaining 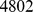 were designated either as pairs in category 5 or pairs in category 1, depending on whether or not they are in pair set two. The above sampling probabilities are illustrated in Figure 2.

#### Simulation process

In each simulation, We generated 50,000 fragment pairs from the pair library with the corresponding set of sampling probabilities. For each sampled pair, we randomly grouped the four ends of the two fragments into two groups and ligated the two ends of each group with probability 0.8. There were three types of ligation products: those that were formed by single fragments with self ligations (self loops), those that were formed by fragment pairs through a single ligation (open loops) and those that were formed by fragment pairs with double ligations (non-self closed loops). For each closed loop (either a self loop or a non-self closed loop), we randomly started from a point and cut along the loop (in one direction) according to a Poisson process until we reached the starting point. For each open loop, we randomly started from a point and cut along the loop (in both directions) according to a Poisson process until we reached the two ends of the loop. In this way we broke the ligation products into many small pieces. Next from each end of the pieces that contained ligation points we read 75 base pairs (bp reads) to get a pair of 75 bp reads. We discarded those read pairs with (at least) one read containing the ligation point to facilitate the following mapping step. Then we mapped each read of a pair to the reference human genome (hg19), and discarded those read pairs with (at least) one read that cannot be uniquely aligned to the genome. Next we considered each alignment (of a read) and identified the fragment in the fragment library from which the corresponding read is derived, and discarded those aligned pairs with both corresponding reads were derived the same fragment, i.e., aligned pairs that were the results of self loops. Finally we associated each aligned pair with a pairing between two different fragments in the fragment library to obtain a data set of counts of the fragment pairs based on their frequencies.

#### Data analysis

We considered the two simulated data sets as a joint simulated data set and applied two models (Two-Step Model and One-Step Model) to the data. For each MCMC algorithm used for MC_DIST or the three-component mixture model of the second step of the Two-Step model, We set the total number of iterations to be 6,000,000 and set the burn-in period to be 1,200,000 to ensure its convergence. For the MCMC algorithm used for the One-Step Model, we set the total number of iterations to be 3,000,000 and set the burn-in period to be 1,500,000 to ensure its convergence. The convergence of the three MCMC algorithms were also confirmed by the respective trace plots of posterior samples. Furthermore, for each MCMC algorithm, we used Raftery and Lewis diagnostic [15] to ensure that the number of iterations and the burn-in were large enough. We also used the Gelman and Rubin’s convergence diagnostic [16] to further check for convergence of the MCMC algorithms.

### Application to ChIA-PET Data

To illustrate our method on real data, we applied the One-Step Model to a two-sample RNA Polymerase II (Pol II) ChIA-PET data set [17]: one of the samples is the breast cancer MCF7 cell line and the other sample is the leukemia K562 cell line. The goal of this experiment and analysis is to identify similarities and differences in chromatin interactions mediated by Pol II in these two cell lines. In particular, special attention is given to identifying chromatin interactions that are unique to MCF7, that is, those that exist in MCF7 but not in K562, category 3 according to our classification scheme in this paper. For this reason, we threshold our data to obtain 9,739 fragment pairs for which the read count for MCF7 is at least 2, whereas the read counts of the corresponding pairs in K562 were used without further thresholding. As such, the read counts for K562 may or may not be at least 2, and further, one would expect more pairs to be classified into category 3 (loops unique to MCF7) than into category 4 (loops unique to K562). For the MCMC algorithm used in the One-Step Model, we set the total number of iterations to be 2,000,000 and set the burn-in period to be 200,000 to ensure its convergence. The convergence of the MCMC algorithms was also confirmed by the respective trace plots of posterior samples. Furthermore, as in the simulation study, we used the Raftery and Lewis diagnostic [15] to ensure that the number of iterations and the burn-in were large enough. We also used the Gelman and Rubin’s convergence diagnostic [16] to further check for convergence of the MCMC algorithms.

## Results

### Simulation Study: Two-step Model

The classification results are summarized in Table 2. We used the Cohen’s kappa [18] to evaluate the classification. Cohen’s kappa is a statistical measure of inter-rater agreement for categorical items. It is between 0 and 1, and larger values indicate better agreement between two raters. It is more robust than simple percent agreement calculation since it takes into account the agreement occurring by chance. Here we treat the truth and the Two-Step Model as two classifiers (raters). Based on the table, the Cohen’s kappa between the Two-Step Model classification and the truth is 0.85. It indicates good agreement [19]. The classification error rate is 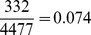. Specifically, the classification error rates for category 0, 1, 2, 3, 4 and 5, are 0 (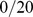), 0.209 (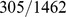), 0.256 (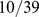), 0.256 (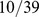), 0.6 (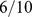) and 0 (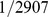), respectively. Note that the classification error rates are quite different for pairs in category 3 (0.256) and pairs in category 4 (0.6). One reason is that half (20) of the pairs of category 3 were designated as true high pairs and all (10) pairs of category 4 were designated as true low pairs in sample one. Thus in sample one, the 20 true high pairs had larger counts and were more easily to be classified correctly than those pairs of category 4 by MC_DIST. Also note that we only simulated 39 (out of the total 40) pairs of category 2 and 39 (out of the total 40) pairs of category 3. The reason is that the two pairs involving fragment 80 (70 bp long) in these two categories were always discarded during the simulation processes as the simulations required to read 75 base pairs from both ends of a ligation product without reaching the ligation point.

**Table 2:** Simulation result: Two-Step Model.

		Classification						Total^a^
		0	1	2	3	4	5	
Actual	0	20^b^	0	0	0	0	0	20
	1	72	1157	80	57	59	37	1462
	2	1	0	29	0	9	0	39
	3	10	0	0	29	0	0	39
	4	0	1	0	0	4	5	10
	5	0	0	0	0	1	2906	2907

a Total number of actual pairs in the row category.

b The number of pairs in the row category that are classified as the pairs in the column category by the Two-Step Model.

We then investigated the performance of the model used in the first step, i.e., MC_DIST. The results are summarized in Table 3. From the table, we see that MC_DIST achieved low type I error rate (almost zero) and high power (0.93) for both data sets. Here the Type I error rate is the proportion of false pairs that are claimed as true pairs by the model, and the power is the proportion of true pairs that are claimed as true pairs by the model. For data set one, the true pairs with high sampling probabilities (pairs in true high set, denoted by the italic numbers) are much more likely to be correctly classified as a true pair than the pairs with low sampling probabilities (pairs in true low set, denoted by the bold numbers), as one would expect. The same tendency was also observed in data set two.

**Table 3:** Simulation result: Step one of the Two-Step Model (MC_DIST).

	Actual					
	−a			+^b^		
	−c	+^d^	Type I error	−	+	Power
data set 1	2707	1	0.0004	**100** e+*4* f	**679** *+776*	*0.933*
data set 2	2724	11	0.004	97	1432	0.937

a Actual false pair.

b Actual true pair.

c Model classified pair as false pair.

d Model classified pair as true pair.

e Pairs in true low set.

f Pairs in true high set.

Next, we investigated the performance of the model used in the second step, i.e., the three-component mixture model. The results is summarized in the subtable of Table 2 formed by the first three columns. Among the 1359 (sum of all numbers in the first three rows of the subtable) pairs that were correctly classified by MC_DIST as true pairs in both samples, 1206 (sum of the three diagonal numbers of the subtable) pairs were classified correctly into their corresponding categories. The percentage of misclassifications is 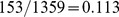. The misclassifications are almost all (152 out of 153) in category 1, i.e., true pairs with no change of interaction intensities. Those 152 pairs were wrongly classified either into category 0 or category 2. The reason is that the pair-specific prior distributions of the mixture weights that we used in the three-component model tend to affect the posterior probabilities of these pairs through the marginal count information.

### Simulation Study: One-step Model

The classification results is summarized in Table 4. Again we used the Cohen’s kappa to evaluate the classification, with the truth and the One-Step Model treated as two classifiers (raters). Based on the table, the Cohens kappa between the One-Step Model classification and the truth is 0.84, which is about the same as the Cohens kappa between the Two-Step Model classification and the truth. The classification error rate is 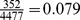, which is slightly higher than the Two-Step Model. Specifically, the classification error rate for category 0, 1, 2, 3, 4 and 5, are 0 (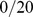), 0.222 (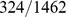), 0.087 (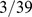), 0.400 (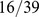), 0.6 (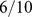) and 0.001 (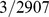), respectively. Compared with the Two-Step Model, the classification error rate for category 2 dropped drastically (from 0.256 to 0.087), although the classification error rate for category 3 increased (from 0.256 to 0.400). Note that four pairs in category 4 were misclassified into category 5. This is because these four pairs were designated as true low pairs in sample two and thus had moderate counts, and they had small marginal counts in sample two and their distances are large. Therefore, it was hard for the One-Step Model to classify them correctly as true pairs in data set two.

**Table 4:** Simulation result: One-Step Model.

		Classification						Total^a^
		0	1	2	3	4	5	
Actual	0	20^b^	0	0	0	0	0	20
	1	127	1138	120	30	26	21	1462
	2	1	0	36	0	2	0	39
	3	16	0	0	23	0	0	39
	4	0	1	1	0	4	4	10
	5	0	2	1	0	0	2904	2907

a Total number of actual pairs in the row category.

b The number of pairs in the row category that are classified as the pairs in the column category by the One-Step Model.

We then investigated the use of One-Step Model as a tool to distinguish true pairs from false pairs in the simulation study. Specifically, for each pair we classified it either as a true pair or a false pair in each sample according to the category to which it was classified by the One-Step Model. The resulted classifications were then compared with those of MC_DIST and we found that the One-Step Model has slightly higher type I error rates and higher powers than MC_DIST. The results are summarized in Table 5.

**Table 5:** Simulation result: Comparison of One-Step Model and MC_DIST as tools to distinguish true pairs from false pairs.

	Actual					
	−a			+^b^		
	−c	+^d^	Type I error	−	+	Power
Sample one	2703 e (2707) f	5 (1)	0.0018 (0.0004)	48 (104)	1511 (1455)	0.969 (0.933)
Sample two	2716 (2724)	19 (11)	0.0069 (0.0040)	53 (97)	1476 (1432)	0.965 (0.937)

a Actual false pair.

b Actual true pair.

c Model classified pair as false pair.

d Model classified pair as true pair.

e Numbers outside of the parentheses represent the numbers of classifications from the One-Step Model.

f Numbers inside the parentheses represent the numbers of classifications from MC_DIST.

### Application to ChIA-PET Data

**Figure 3:**
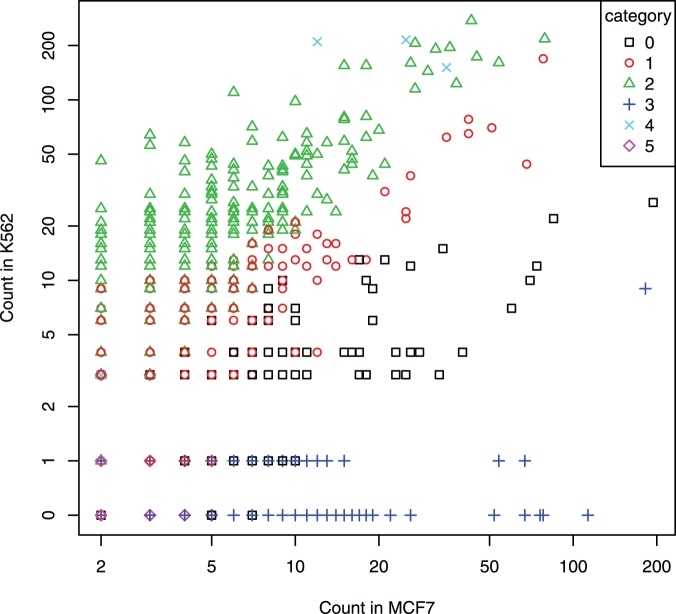
One-Step Model result on the real data. The *x*-axis is the count in the cell line MCF7 and the *y*-axis is the count in the cell line K562. Note that the entire *x*-axis and the *y*-axis are drawn according to the log-scale to accommodate the large range of counts. To include those pairs with count 0 in the cell line K562, we replaced the 0’s by 0.5’s and changed the label from 0.5 to 0 on the *y*-axis. Each point represents a fragment pair and its color and shape indicates the category it was classified into by the One-Step Model.

We analyzed the data using the One-Step Model because the number of fragment pairs (9739) is relatively small and because the model is considered to be more efficient by taking the uncertainty in loop detection into consideration. The two data sets were normalized using the number of raw reads in each experiment. The proportions of fragment pairs that were classified into category 0, 1, 2, 3, 4 and 5 (see Table 1 for the descriptions of these 6 categories) are: 0.009 (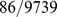), 0.121 (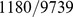), 0.040 (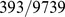), 0.100 (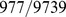), 0.001 (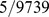), and 0.729 (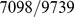), respectively. Note that 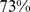 of them are classified as false pairs in both cell lines, which is not surprising as there are many that have small counts (Figure 3). For pairs with true interaction in at least one of the cell lines, there are a number of examples that show consistent results with what have been documented in the literature. For example, the chromatin interaction for a pair on chromosome 2 (fragment 11670480–11672120 and fragment 11681427–11683543), with a count of 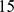 in cell line MCF7 and a count of 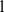 in K562, was classified into category 3. This interaction is around the gene *GREB1*, which is known to be a true interaction in the MCF7 cell line validated by 3C experiment [17], but not in the K562 cell line, that is, the interaction is unique to MCF7, the type of interactions that is of particular interest.

To gain a more comprehensive and greater understanding of the results for all pairs considered, in Figure 3, we plotted the count of fragment pair for MCF7 (*x*-axis; log-scale) versus the count of the same fragment pair for K562 (*y*-axis; log-scale). Results for categories 0, 1, and 2 (for which the interactions are true ones for both cell lines) are intuitively sensible. All the green triangles (category 2, indicating K562 has higher looping intensity than MCF7) are above the diagonal line, whereas the black squares (category 0, in which MCF7 is inferred to have higher looping intensity than K562) are below the diagonal line, with a couple exceptions. On the other hand, pairs that are marked as red circles (category 1, in which MCF7 and K562 have same looping intensity) are scattered around the diagonal line. Of course, other than the counts themselves, marginal counts and distance to TFBS and TSS also contribute to the classification, whose effects are displayed on Figure 3 as well. For example, the few pairs for which the read counts for MCF7 are slightly smaller than the corresponding counts in K562 (differences in counts are all 1) but are classified into category 0 are primarily due to the marginal counts in MCF7 being much larger than the corresponding marginal counts in K562. As another example, pairs with the same counts in both cell lines may be classified into different categories (symbols overlapping one another in Figure 3), for which not only different marginal counts, but also different distances, may play big roles. One particular instance is noteworthy: there are multiple pairs in which the count for MCF 7 is 2 whereas the count for K562 is 3, but these pairs are classified into five different categories - 0, 1, 2, 4, and 5. For pairs classified into category 3 (unique loops for MCF7), the counts for MCF7 are indeed much larger than the corresponding counts for K562, almost all being at most 1 (blue crosses in Figure 3). These results answered the scientific question that there are indeed chromatin interactions that are unique to MCF7, and the results are consistent with those in the literature, such as the *GREB1* gene discussed above. Finally, there are only a few pairs that are classified into category 4 (loops unique to K562). For three of these pairs, although the counts for MCF7 are moderate (but relatively much smaller compared to the counts for K562), the distances (two of them) can be much larger than a typical distance, contributing to the loops for MCF7 classified as false. The other two pairs both have a count of 2 for MCF7 and 3 for K562 (overlapping with other pairs in Figure 3 as mentioned above) and are classified as such mainly due to their marginal counts for K562 being much larger (about 300) than the corresponding counts for K562 (about 60). The number of pairs classified into category 4 being so small is as expected given our focus on identifying chromatin interactions that are unique to MF7, and hence the thresholding scheme. Had we treated the two cell lines in a equal footing without thresholding the MCF7 counts, we would have seen a more symmetrical plot where there would be many more pairs classified into category 4 and shown along the *y*-axis where the MCF7 counts are 0 and 1.

**Figure 4:**
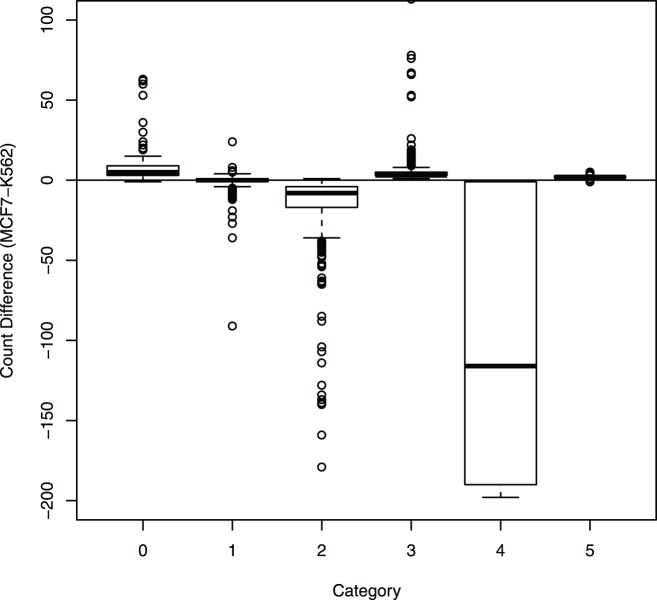
Boxplot of the count differences for each category for the real data. The *x*-axis is the category that the fragment pair was classified into. The *y*-axis is the count in the cell line MCF7 minus the count in the cell line K562 for the fragment.

To further illustrate the different patterns of count differences in these categories, we provide, in Figure 4, a boxplot of the differences in counts (MCF7 - K562) for pairs classified to each category. One can be seen clearly that for categories 0 and 3, almost all the differences are above 0, whereas for categories 1 and 4, the differences are below 0. For the two categories (2 and 5) where interactions in neither cell lines is a true one or where the interaction intensities for the two cell lines are inferred to be the same, the differences scatter around 0.

## Discussion

Chromatin interactions mediated by a protein of interest are of great scientific interest, as chromatin interactions can have different intensities in different cells and such differences can be associated with a disease. A recent technique, Chromatin Interaction Analysis by Paired-End Tag Sequencing (ChIA-PET), was developed to study chromatin interactions mediated by a protein of interest in a genome-wide manner. Thus, a question of interest is how to detect differential chromatin interactions based on two sample ChIA-PET (count) data.

In this paper, we proposed a Two-Step Model and a One-Step Model to identify chromatin interactions with different intensities in two samples. Both models classify the observed fragment pairs into six categories. The Two-Step Model consists of two models: the MC_DIST (used in the first step) and a three-component mixture model (used in the second step), and the One-Step Model is a six-component mixture model. All three models are cast into Bayesian frameworks to take the data dependency and the extent to which a fragment pair is related to the DNA loci of interest into account. Since our goal is to identify all differential chromatin interactions regardless of whether such interactions are cis-chromosomal or trans-chromosomal [20], no distinction is being made in this paper. However, our method can be modified to accommodate such a distinction if desired. All the models have been included in an R-package *MDM* which is available at http://www.stat.osu.edu/~statgen/SOFTWARE/MDM. In the package, there are three main functions: one for MC_DIST, one for the three-component mixture model and one for the One-Step Model.

We evaluated the proposed methodology using a large scale simulation study. We further applied the One-Step Model to analyzing a two-sample ChIA-PET data set to characterize their similarities and differences in chromatin interactions, focusing on interactions that are unique to MCF 7. The results of the simulation study showed that both methods performed well, with low type I error rates and reasonably good power. The results from the real data analysis shows that the inferences appear to be sensible based on known biological knowledge and data characteristics. By making use of the data more efficiently, the One-Step Model is expected to outperform the Two-Step model. However, it is more computational intensive than the Two-Step Model. When the number of MCMC iterations is set to be the same, the computing time of the One-Step Model is about 40% longer than that of the Two-Step Model in the simulation study. We expect that for larger cut-off value of the counts (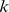 in the models), the Two-Step Model is even more computationally efficient than the One-Step model, since many pairs with small counts (less than 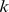) are excluded in the first step and only a subset of the observed data is considered in the second step.

To show the benefits of incorporation of data dependency and the extent to which a fragment pair is related to the DNA loci of interest, we modified the three-component mixture model used in the second step of the Two-Step Model and the One-Step Model so that the mixture weights are common for all pairs and assumed that the mixture weights of each modified model follow a uninformative prior distributions, i.e., the uniform distribution over the corresponding simplex. Then we ran the modified models on the simulated data and compared the results with those of the original models. The modified three-component mixture model induced an unacceptable misclassification rate for pairs in category 0 (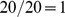) or category 2 (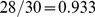), although it committed only one misclassification for the 1309 pairs in category 1. The modified On-Step Model also performed poorly, It classified almost all the pairs which are true pairs in both samples as pairs with no change of interaction intensities (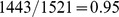), and the Cohen’s kappa for the agreement between the actual and the classification is only 0.54, indicating a moderate agreement [19].

Although the proposed models perform well in both the simulation study and the application, there is still room for improvement. For example, we can incorporate the ChIP-Seq data and RNA-Seq data into the One-Step Model and MC_DIST to improve the calculation of distance information for pairs. In these two models, we define the distance for a fragment pair as the sum of two distances: the distance between the midpoint of one fragment and its closest gene TSS and the distance between the midpoint of the other fragment and its closest transcription factor binding site (TFBS), where the transcription factor is the protein of interest. To calculate the distances, we use the gene annotation to locate the gene TSS’s and a list of known TFBS’s (a union of TFBS’s identified in multiple experiments on multiple cell types) to locate TFBS’s. However, for some fragment pairs, such gene and TFBS might be irrelevant to the ChIA-PET study, thus including such distances into MC_DIST might lead to wrong classifications. We can improve the performance by considering binding sites of, and genes that are regulated by, protein of interest. This would require an integrated analysis of the ChIA-PET, RNA-Seq and ChIP-Seq data.

## References

[1] Gong F, Sun L, Wang Z, Shi J, Li W, et al (2011) The bcl2 gene is regulated by a special at-rich sequence binding protein 1-mediated long range chromosomal interaction between the promoter and the distal element located within the 3-utr. Nucleic acids research 39: 4640–4652. 21310710 10.1093/nar/gkr023 PMC3113567

[2] Guo Y, Monahan K, Wu H, Gertz J, Varley KE, et al (2012) Ctcf/cohesin-mediated dna looping is required for protocadherin *α* promoter choice. Proceedings of the National Academy of Sciences 109: 21081–21086. 10.1073/pnas.1219280110 PMC3529044 23204437

[3] Decker KF, Zheng D, He Y, Bowman T, Edwards JR, et al (2012) Persistent androgen receptormediated transcription in castration-resistant prostate cancer under androgen-deprived conditions. Nucleic acids research 40: 10765–10779. 23019221 10.1093/nar/gks888 PMC3510497

[4] Kohwi-Shigematsu T, Kohwi Y, Takahashi K, Richards HW, Ayers SD, et al (2012) Satb1-mediated functional packaging of chromatin into loops. Methods 58(3): 243–254. 22782115 10.1016/j.ymeth.2012.06.019 PMC4029128

[5] Wang Q, Li W, Zhang Y, Yuan X, Xu K, et al (2009) Androgen receptor regulates a distinct transcription program in androgen-independent prostate cancer. Cell 138: 245–256. 19632176 10.1016/j.cell.2009.04.056 PMC2726827

[6] Jiang H, Peterlin BM (2008) Differential chromatin looping regulates cd4 expression in immature thymocytes. Molecular and cellular biology 28: 907–912. 18039856 10.1128/MCB.00909-07 PMC2223376

[7] Junier I, Dale RK, Hou C, Képès F, Dean A (2012) Ctcf-mediated transcriptional regulation through cell type-specific chromosome organization in the *β*-globin locus. Nucleic acids research 40: 7718–7727. 22705794 10.1093/nar/gks536 PMC3439919

[8] Jing H, Vakoc CR, Ying L, Mandat S, Wang H, et al (2008) Exchange of gata factors mediates transitions in looped chromatin organization at a developmentally regulated gene locus. Molecular cell 29: 232–242. 18243117 10.1016/j.molcel.2007.11.020 PMC2254447

[9] Fullwood MJ, Liu MH, Pan YF, Liu J, Xu H, et al (2009) An oestrogen-receptor-alpha-bound human chromatin interactome. Nature 462: 58–64. 19890323 10.1038/nature08497 PMC2774924

[10] Dekker J, Rippe K, Dekker M, Kleckner N (2002) Capturing Chromosome Conformation. Science 295: 1306–1311. 11847345 10.1126/science.1067799

[11] Lieberman-Aiden E, van Berkum NL, Williams L, Imakaev M, Ragoczy T, et al (2009) Comprehensive mapping of long-range interactions reveals folding principles of the human genome. Science 326: 289–293. 19815776 10.1126/science.1181369 PMC2858594

[12] Niu L, Lin S (2013) Statistical modeling and analysis of chromatin interactions mediated by a protein. Technical Report 876, Department of Statistics, The Ohio State University.

[13] Rousseau M, Fraser J, Ferraiuolo M, Dostie J, Blanchette M (2011) Three-dimensional modeling of chromatin structure from interaction frequency data using Markov chain Monte Carlo sampling. BMC Bioinformatics 12: 414. 22026390 10.1186/1471-2105-12-414 PMC3245522

[14] Obinata D, Takayama Ki, Urano T, Murata T, Kumagai J, et al (2012) Oct1 regulates cell growth of lncap cells and is a prognostic factor for prostate cancer. International Journal of Cancer 130: 1021–1028. 21387309 10.1002/ijc.26043

[15] Raftery AE, Lewis SM (1995) The number of iterations, convergence diagnostics and generic metropolis algorithms. In: Gilks WR, Spiegelhalter DJ, Richardson S, editors. Practical Markov Chain Monte Carlo. Chapman and Hall. 115–130.

[16] Gelman A, Rubin DB (1992) Inference from iterative simulation using multiple sequences. Statistical Science 7: 457–472.

[17] Li G, Ruan X, Auerbach RK, Sandhu KS, Zheng M, et al (2012) Extensive promoter-centered chromatin interactions provide a topological basis for transcription regulation. Cell 148: 84–98. 22265404 10.1016/j.cell.2011.12.014 PMC3339270

[18] Cohen J (1960) A coefficient of agreement for nominal scales. Educational and psychological measurement 20: 37–46.

[19] Landis JR, Koch GG (1977) The measurement of observer agreement for categorical data. Biometrics 33: 159–174. 843571

[20] Handoko L, Xu H, Li G, Ngan CY, Chew E, et al (2011) Ctcf-mediated functional chromatin interactome in pluripotent cells. Nature genetics 43: 630–638. 21685913 10.1038/ng.857 PMC3436933

